# A cluster randomised trial of educational messages to improve the primary care of diabetes

**DOI:** 10.1186/1748-5908-6-129

**Published:** 2011-12-16

**Authors:** Robbie Foy, Martin P Eccles, Susan Hrisos, Gillian Hawthorne, Nick Steen, Ian Gibb, Bernard Croal, Jeremy Grimshaw

**Affiliations:** 1 Leeds Institute of Health Sciences, Charles Thackrah Building, University of Leeds, 101 Clarendon Road, Leeds LS2 9LJ, UK; 2 Institute of Health and Society, Newcastle University, The Baddiley-Clark Building, Richardson Road, Newcastle upon Tyne, NE2 4AX, UK; 3 Newcastle NHS Primary Care Trust, Newcastle Diabetes Centre, Newcastle General Hospital, Newcastle upon Tyne, NE4 6BE, UK; 4 Clinical Biochemistry, Newcastle Upon Tyne Hospitals NHS Foundation Trust, Royal Victoria Infirmary, Queen Victoria Road, Newcastle upon Tyne, NE1 4LP, UK; 5 Department of Clinical Biochemistry, Aberdeen University Medical School, Polwarth Building, Aberdeen, AB25 2ZD, UK; 6 Clinical Epidemiology Program, Ottawa Health Research Institute, 725 Parkdale Avenue, Ottawa, ON K1Y 4E9, Canada

## Abstract

**Background:**

Regular laboratory test monitoring of patient parameters offers a route for improving the quality of chronic disease care. We evaluated the effects of brief educational messages attached to laboratory test reports on diabetes care.

**Methods:**

A programme of cluster randomised controlled trials was set in primary care practices in one primary care trust in England. Participants were the primary care practices' constituent healthcare professionals and patients with diabetes. Interventions comprised brief educational messages added to paper and electronic primary care practice laboratory test reports and introduced over two phases. Phase one messages, attached to Haemoglobin A1c (HbA1c) reports, targeted glycaemic and cholesterol control. Phase two messages, attached to albumin:creatinine ratio (ACR) reports, targeted blood pressure (BP) control, and foot inspection. Main outcome measures comprised practice mean HbA1c and cholesterol levels, diastolic and systolic BP, and proportions of patients having undergone foot inspections.

**Results:**

Initially, 35 out of 37 eligible practices participated. Outcome data were available for a total of 8,690 patients with diabetes from 32 practices. The BP message produced a statistically significant reduction in diastolic BP (-0.62 mmHg; 95% confidence interval -0.82 to -0.42 mmHg) but not systolic BP (-0.06 mmHg, -0.42 to 0.30 mmHg) and increased the odds of achieving target BP control (odds ratio 1.05; 1.00, 1.10). The foot inspection message increased the likelihood of a recorded foot inspection (incidence rate ratio 1.26; 1.18 to 1.36). The glycaemic control message had no effect on mean HbA1c (increase 0.01%; -0.03 to 0.04) despite increasing the odds of a change in likelihood of HbA1c tests being ordered (OR 1.06; 1.01, 1.11). The cholesterol message had no effect (decrease 0.01 mmol/l, -0.04 to 0.05).

**Conclusions:**

Three out of four interventions improved intermediate outcomes or process of diabetes care. The diastolic BP reduction approximates to relative reductions in mortality of 3% to 5% in stroke and 3% to 4% in ischaemic heart disease over 10 years. The lack of effect for other outcomes may, in part, be explained by difficulties in bringing about further improvements beyond certain thresholds of clinical performance.

**Trial Registration:**

Current Controlled Trials, ISRCTN2186314.

## Background

Despite continuing improvements in the delivery and outcomes of care for people with diabetes [1-3] there is still evidence of substantial inappropriate variations [4-8]. A systematic review of quality improvement strategies for patients with type 2 diabetes indicated small to modest effects on glycaemic control [9]. Much work remains to be done in developing and evaluating many quality improvement strategies given that they are often resource-intensive and the difficulties in reliably identifying their 'active ingredients' [10].

Across a range of different targeted clinical behaviours and contexts, in general reminders delivered to healthcare professionals consistently improve performance [11], an effect also apparent with point-of-care computer reminders [12]. However, the optimal configuration of such reminders is still unclear. One approach that offers the potential advantages of simplicity and sustainability is attaching brief educational messages to the results of tests ordered in the expectation that the healthcare professional who ordered the test will read and act on the message when the result is delivered back to the practice. Attached to radiology reports ordered from primary care, such an intervention reduced requests for targeted x-rays without compromising quality of referrals [13]. Messages attached to test reports also reduced primary care laboratory test requests [14].

However, previous research on interventions to change test ordering-related behaviour has largely focused on either decreasing the overall volume of tests ordered (in the implicit belief that some are unnecessary) or specifically decreasing the number of inappropriate tests ordered; 47 out of 49 studies in one review focused on such reductions [15]. Less is known about the effectiveness of test ordering messages in improving wider aspects of clinical management to promote evidence-based care.

In one primary care trust in the north east of England, we evaluated the effects on the care provided for patients with type 2 diabetes of educational prompts attached to laboratory test reports and aimed at increasing evidence-based clinical practice.

## Methods

### Study design

The study used a cluster randomised controlled trial design, described in detail elsewhere [16], with primary care practices as the unit of randomisation.

### Participants, setting and context

The study participants were the clinicians--general practitioners (GPs) and nurses--working in those primary care practices in Newcastle upon Tyne that used the laboratory services of the Newcastle Hospitals NHS Trust (now the Newcastle Hospitals NHS Foundation Trust). Outcomes were assessed on all those patients registered with each practice and with a diagnosis of diabetes whose care was undertaken either by the practice or shared between the practice and hospital.

The study started in late 2005, one year after the advent of performance-related funding for primary care physicians [17]. Through the Quality and Outcomes Framework (QOF), practices earned points for achieving targets across a range of clinical and organisational indicators. Performance against these targets can generate up to 40% of practice income. The QOF initially included 18 diabetes indicators that were extended or modified over subsequent years [18].

### Interventions

The interventions were four brief educational messages, typically of less than 30 words, added to the returned results of laboratory tests ordered by clinicians on patients with diabetes cared for in intervention practices. The educational messages were developed by a multi-disciplinary group that included clinical representatives from primary care, secondary care, laboratory services, and the research team. The messages gave succinct evidence-based, educational information regarding appropriate patient management congruent with the local diabetes clinical guideline (Table 1). The message topics were selected because of their clinical importance, and the feasibility of being able to measure improvements in relevant outcomes from routinely held data in primary care practices.

**Table 1:** Content of the laboratory test messages

Study phase	Type and attachment of message	Content of message
Phase One	Unconditional; attached to all cholesterol reports	For type 2 diabetes and age ≥ 40 yrs: on simvastatin 40 mg? See [local guideline] for detail and exclusions

	Conditional; attached to all HbA1c reports	If HbA1c < 6.5% 'Within target for type 2 diabetes' If HbA1c 6.5 to 7.0% 'For type 2 diabetes, consider increasing oral therapy' If HbA1c 7.0 to 8.0% 'If type 2 diabetes: on max oral therapy, *e.g*., Metformin 1G BD + gliclazide 160 mg BD?' If HbA1c > 8.0% 'If type 2 diabetes, consider insulin if on max oral Rx, *e.g*., Metformin 1G BD + gliclazide 160 mg BD'

Phase Two	Unconditional; attached to all albumin: creatinine ratio (ACR) test reports	'Newcastle Diabetes Guideline Footcare: all patients annual review of sensation, pulses, footwear education'

	Conditional; attached to all ACR test reports	If ACR above 2.5 'If confirmed microalbuminuria: aim for BP < 130/80 in type 2 diabetes' If ACR below 2.5 'If no microalbuminuria: aim for BP control < 140/80 in type 2 diabetes'

The interventions were introduced in two phases. In phase one (December 2005), messages were attached to electronic and paper Haemoglobin A1c (HbA1c) test reports. The messages were of two types. The first message related to glycaemic control, was conditional on the HbA1c level, and gave advice about appropriate treatment. The second type of message gave a non-conditional message relating to cholesterol control.

In phase two (October 2006), messages were attached to albumin:creatinine ratio (ACR) test reports and were also of two types. The first message related to blood pressure (BP) control, was conditional on the ACR level, and gave advice on target BP levels for patients with and without a diagnosis of microalbuminuria. The second message related to foot inspection and was non-conditional.

### Intervention fidelity

We contacted at least two practices in each of the four study arms at six-month intervals (different practices each time) to check whether practices were receiving their allocated messages, and that the messages continued over the intervention period as planned

### Randomisation

In each of two phases primary care practices were randomised twice to receive or not each of two educational messages. Thus, in each phase 25% of practices received both messages, 25% each received one of the messages, and 25% received no intervention. For phase one, primary care practices were randomised twice to receive or not the glycaemic educational messages and to receive or not the cholesterol educational messages. Randomisation was stratified using existing routine data by both the number of patients with diabetes per practice (using a median split of 200) and the proportion of patients with an HbA1c of 7.4% or less (grouped by less than 60%, 60% to 70%, and over 70%) [17].

For phase two, ten months later, practices were randomised twice to receive or not the foot inspection reminder message and to receive or not the BP educational messages. On this occasion randomisation was stratified by practice QOF scores for recorded foot examination (median split of 2.85 points out of 3) and the proportion of patients with a record of BP of 145/85 mmHg or less (using median split of 74.8%). All randomisations were conducted independently by a statistician using numbers randomly generated by computer.

### Outcomes

The main outcomes were the primary care practices' mean levels of HbA1c, cholesterol and BP, and numbers of patients with recorded foot inspections in the previous calendar month. Other analysed outcomes were: the number of patients within target ranges for HbA1c, cholesterol, and BP; the number of HbA1c, cholesterol, and ACR tests requested (standardised for practice size); and mean practice BP levels for patients with and without recorded microalbuminuria (operationalised as a record of two or more consecutive ACRs of 2.5 or greater).

### Data collection

National Health Service (NHS) staff collected coded data from practice computer systems using customised electronic queries. They were not blinded to group assignment. They removed patient identifiers before the transfer of data to the research team. For phase one interventions, there were 24 and 34 months of pre- and post-intervention outcome data, respectively, whilst for phase two interventions, there were similarly 34 and 24 months of data. This produced multiple observations for patients over the study. In general, practices routinely collect and code patient data that contribute to the calculation of scores for the QOF [17]. Our data were similar (though not identical) to these and within QOF practices are subject to independent scrutiny of their data for accuracy and completeness, thereby allowing a considerable degree of confidence in data quality, though we did not independently assess this.

### Sample size

The sample size calculations, based on methods described by Donner *et al*. [19], were undertaken using a programme developed by Campbell *et al*. [20]. They were based upon the following assumptions: 34 participating practices each with a mean number of 62 patients with diabetes; a significance level of 5%; 80% power; and an intra-class correlation coefficient (ICC) of 0.2 for process measures (based upon recording of blood pressure and HbA1c for an earlier trial [21]) and 0.05 for intermediate outcomes [22]. With these assumptions, we would be able to detect a 21% improvement (from 55% to 76%) in a binary outcome measure (*e.g*., foot examination) and an effect size of 0.25 in a continuous outcome measure. The latter represents changes of 0.36% in mean HbA1c, 0.27 mmol/L in mean cholesterol, 4.98 mmHg in mean systolic BP, and 2.55 mmHg in mean diastolic BP.

### Analysis

The analytic strategy differed from that given in the published protocol [16]. First, we were able to run the study over a longer period than initially anticipated, giving us the opportunity to capitalise on the fact that we could collect data over an extended period of time. An interrupted time series analysis approach allowed use of all the available data rather than reducing them to single observations per patient pre- and post-intervention [23]. Second, analyses were also adjusted to take account of factors used to stratify randomisation.

Only observations made after a patient was first diagnosed with diabetes were included in the analysis. For continuous dependent variables (BP, HbA1c and cholesterol values), a three-level, multilevel model incorporating random variation between practices, random variation between patients within practices, and random variation between repeated measures within patients was used to investigate the impact of the interventions. The following fixed effects were investigated: general trends in the dependent variable over time; a difference between intervention and control practices across the entire period of investigation; and a difference between observations made prior to the relevant intervention and those made after. Fitting an interaction between the last two effects then provided an estimate of the effect of the intervention.

The continuous variables were also dichotomised: patients were categorised as being above (not controlled) or below (controlled) target thresholds described in the educational messages. These binary variables were analysed using a three-level, multilevel model as described above except that a binomial error structure was assumed for the random variation at the lowest level of the model.

To investigate the effect of the intervention on rates of foot inspection and rates of test ordering, the dependent variable was the number of patients for whom a foot inspection or test result was recorded in a practice in a calendar month. This was analysed using a two-level, negative binomial regression model with months nested within practices. The log of the number of patients with diabetes in the practice during the month was included as an offset. The effects of the interventions were then estimated using the approach described above for the continuous variables.

### Ethical approval

The study was approved by the Newcastle and North Tyneside Research Ethics Committee (Reference number 05/Q0905/95).

## Results

**Figure 1:**
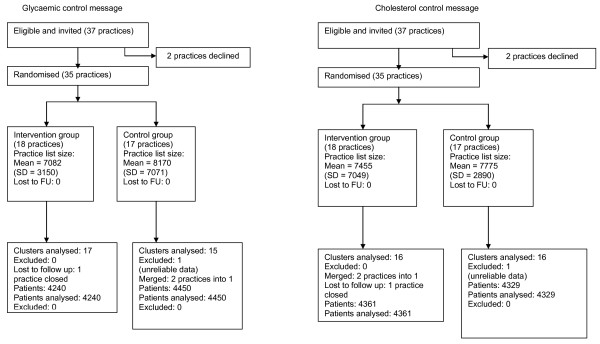
**CONSORT flow charts for Phase One**.

**Figure 2:**
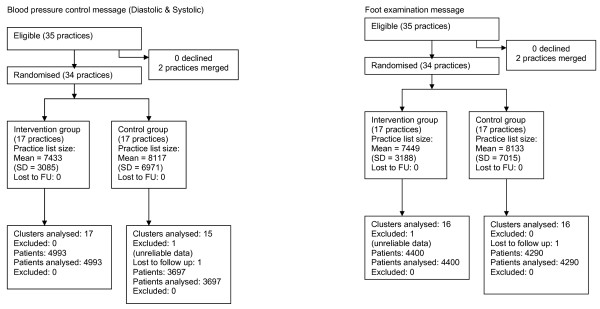
**CONSORT flow charts for Phase Two**.

Of 37 eligible practices, 35 agreed to participate and two declined. By the time of outcome data collection, two practices had merged into one (both cholesterol message-only practices at first randomisation, whilst one received both messages and one foot inspection messages only at second randomisation) and a further practice had closed (glycaemic and cholesterol messages only). Following inspection, clinical data were considered unusable for one control practice (which appeared to be using HbA1c as a diagnostic as well as monitoring test) and it was therefore excluded from the analysis. Thus outcome data were available for 32 practices. We identified a total of 8,690 patients with diagnoses of type 2 diabetes made before or during the study period. Table 2 shows the baseline characteristics of study practices and their patients. The CONSORT diagrams (Figures 1 and 2) summarise the flows of recruitment, participation, and analysis.

**Table 2:** Pre-intervention characteristics of intervention and control groups.

Trial intervention	Pre-intervention characteristics	Intervention group	Control group
**Glycaemic control**	**Practice factors**		

	Number	18	16

	Median number of partners	4.5	4

	Mean (SD) list size	7,082 (3150)	8,170 (7071)

	**Patient factors**		

	Mean (SD) number in practice	196 (108)	205 (120)

	Mean age (years, SD)	65 (14)	63 (14)

	Proportion men (%)	52	53

	Mean (last recorded) HbA1c (SD)	7.4 (1.4)	7.4 (1.4)

**Cholesterol control**	**Practice factors**		

	Number	18	16

	Median number of partners	4	4

	Mean (SD) list size	7,455 (7049)	7,775 (2890)

	**Patient factors**		

	Mean (SD) number in practice	188 (109)	213 (117)

	Mean age (years, SD)	64 (14)	64 (14)

	Proportion men (%)	52	53

	Mean (last recorded) cholesterol (mmol/l)	4.7 (1.2)	4.5 (1.1)

**Blood pressure control**	**Practice factors**		

	Number	17	17

	Median number of partners	5	4

	Mean (SD) list size	7,433 (3085)	8,117 (6971)

	**Patient factors**		

	Mean (SD) number in practice	239 (128)	200 (116)

	Mean age (years, SD)	65 (14)	63 (14)

	Proportion men (%)	53	53

	Mean (last recorded) systolic BP (mmHg)	145	147

	Mean (last recorded) diastolic BP (mmHg)	80	80

**Foot examination**	**Practice factors**		

	Number	17	17

	Median number of partners	4	4

	Mean (SD) list size	7,449 (3188)	8,133 (7015)

	**Patient factors**		

	Mean (SD) number in practice	223 (105)	218 (139)

	Mean age (years, SD)	64 (14)	64 (14)

	Proportion men (%)	51	54

	Proportion with a recorded foot inspection in the previous 15 months (%)	2536 (69)	2761 (78)

We checked with 18 practices as to whether they were receiving allocated interventions. For the eight various possible combinations of the four intervention messages, at least two practices per combination confirmed receipt of the messages as allocated. There were no cases of practices receiving an unallocated message.

The mean intra-cluster correlations (ICCs) at baseline were 0.03 (95% CI: 0.02, 0.05) for HbA1c, 0.04 (0.02, 0.07) for systolic BP, 0.02 (0.01, 0.03) for diastolic BP, and 0.06 (0.03, 0.1) for cholesterol. For foot inspection, the corresponding figures were 0.34 (0.28, 0.53), reflecting large systematic differences between practices in the recording of this variable.

**Figure 3:**
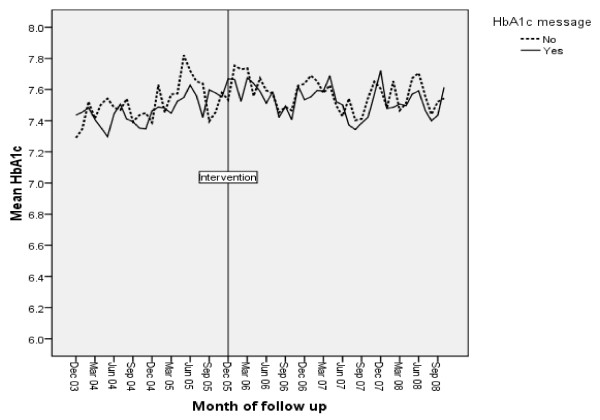
**Mean HbA1c during period of study by calendar month**.

**Figure 4:**
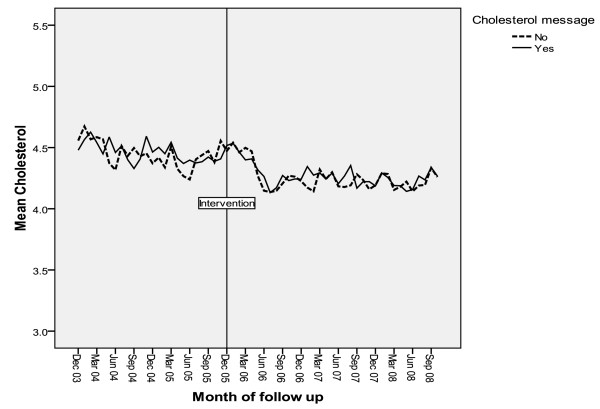
**Mean HbA1c during period of study by calendar month**.

**Figure 5:**
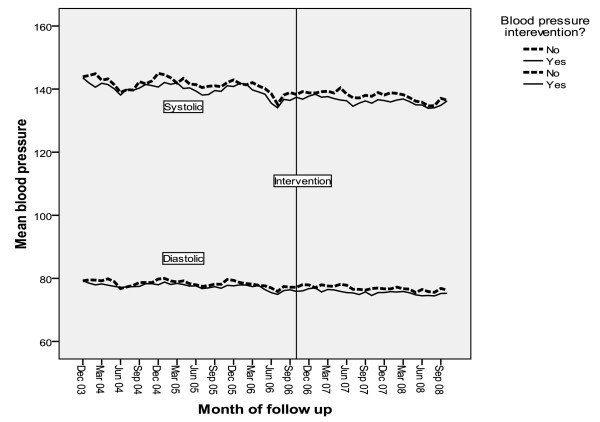
**Mean systolic and diastolic BP during period of study by calendar month**.

In general, the clinical values were already reasonable, with baseline control group mean values of HbA1c of 7.4%, cholesterol of 4.5 mmol/l, and BP of 147/80 mmHg. Figures 3, 4 and 5 show the intervention and control trends in their values over the study period. Table 3 shows the estimated effects of the interventions based on the multilevel models.

**Table 3:** Estimated impact of the interventions.

Outcome	Effect of intervention			
	**Parameter**^**†**^	**Estimate**^**‡**^	**95% CI**	

**Glycaemic control message**				

HbA1c (%)*	mean	0.01	-0.03	0.04

HbA1c within target level of control (less than 6.35)	OR	0.94	0.87	1.03

Number of HbA1c tests being ordered in a calendar month	IRR	1.06	1.01	1.11

**Cholesterol control message**				

Cholesterol (mmol/l)*	mean	0.01	-0.04	0.05

Cholesterol within target level of control (5 mmol/l or less)	OR	1.01	0.92	1,11

Number of a cholesterol tests being ordered in a calendar month	IRR	1.00	0.95	1.05

**Blood pressure control message**				

Systolic blood pressure (mm Hg): all patients*	mean	-0.06	-0.41	0.30

Systolic blood pressure (mm Hg): patients with micro-albuminuria from 21 practices	mean	1.38	0.33	2.42

Diastolic blood pressure (mm Hg): all patients*	mean	-0.52	-0.73	-0.32

Diastolic blood pressure (mm Hg): patients with micro-albuminuria from 21 practices	mean	0.61	0.03	1.19

Blood pressure within target level of control (less than or equal to 140/80): all patients	OR	1.05	1.00	1.10

Blood pressure within target level of control (less than or equal to 130/80): patients with micro-albuminuria	OR	0.88	0.78	0.99

**Foot inspection message**				

Number of patients in practice for whom a foot inspection was recorded in a calendar month*	IRR	1.26	1.18	1.36

†Notes

• mean: change in mean that can be attributed to the intervention

• OR: odds ratio (relative increase in odds of stated outcome)

• IRR: incidence rate ratio (relative increase in likelihood of stated outcome)

‡ Estimates adjusted for strata used when randomising practices.

*Main outcome measure

There was no intervention effect on HbA1c (increase 0.1%; 95% CI -0.03, 0.04) or good glycaemic control (HbA1c less than 6.35%; OR 0.94; 95% 0.87, 1.03). However, the intervention produced an increase in the likelihood of a test being ordered (IRR 1.06; 95% CI 1.01, 1.11).

There was no intervention effect on mean cholesterol levels, whether or not cholesterol was within target range or cholesterol testing rates.

For systolic BP, there was a mean annual reduction of 1.59 (95% CI 1.49, 1.69) mmHg during the study period, but no intervention effect. For diastolic BP, there was a mean annual reduction of 0.92 (95% CI 0.81, 1.02) mmHg during the study period, and a statistically significant reduction (-0.52 (95% CI -0.73, -0.32) mmHg in intervention practices. The intervention increased the odds of patient BP being controlled at or under 140/80 (OR 1.05 (95% CI 1.00, 1.10).

For the BP analyses, we also considered the possibility of a delayed effect given the time taken for clinical review and treatment titration of patients with raised BP. We therefore undertook an exploratory *post hoc *analysis, fitting a delayed effect to the model that assumed a gradual increase in the intervention effect over a four month period. This analysis found no evidence of a delayed effect.

For foot examination, there was an increased likelihood of a recorded foot inspection (IRR 1.26; 95% CI 1.18, 1.36) in intervention practices.

The BP analyses planned for patients with microalbuminuria were constrained by missing data because ACR results were only available from a subset of 21 of the 32 practices included in the final analysis. In these 21 practices (comprising 14 and seven of the intervention and control groups, respectively) there were 5,765 patients who each contributed between one and 108 BP measurements over the study period (79,135 measurements in total). There were 1,019 patients with two or more consecutive ACR values greater than 2.5 (18,358 BP measurements in total corresponding to these patients). In patients with microalbuminuria, mean systolic BP increased by 1.38 (0.33, 2.42) mmHg, whilst there was a non-significant increase in diastolic BP of 0.61 (-0.03, 1.19) mmHg.

The intervention reduced the odds of a patient with microalbuminuria having their blood pressure controlled at or under 130/80 mmHg (OR 0.88; 95% CI 0.78, 0.99).

## Discussion

Three of the four educational messages accompanying laboratory test reports influenced clinical behaviour for two primary outcomes (BP and foot inspections) and one secondary outcome (HbA1c testing). Patient endpoints improved, with a small decrease in diastolic BP and an increase in the proportion of patients with controlled BP, and processes of care changed, with increased likelihoods of recorded foot inspections and HbA1c testing. Where they occurred, the effects appeared immediately after initiation of the messages. Once established, given its automated nature, this intervention is likely to be easily sustainable. These effects were achieved against a background of improving performance accelerated by financial incentives [3]; the average overall percentage level of achievement for the QOF diabetes indicators had improved over 2005 to 2009 from 93.2% to 98.4% in England and from 97.9% to 99.7% in Newcastle upon Tyne [18]. Furthermore, in a recent comparative audit, the primary care trust in Newcastle was ranked first out of 152 trusts in England in having the highest percentage of people with diabetes with blood pressure less than 145/85 mmHg [24]. This makes the achievement of improving BP levels and control particularly notable. Against such background levels of achievement in relation to the QOF (94% for checking peripheral pulses in 2009 in Newcastle upon Tyne), it would also be surprising if the increased likelihood of foot inspection was merely attributable to better recording.

Although how well foot inspection was performed is unknown, the observed change is likely to represent a true improvement in care. This is important given the recognised value of multiple risk factor reduction, including for vascular endpoints, in type 2 diabetes [25].

Previous studies of test-ordering messages have demonstrated reductions in request rates [12-14]. This is one of a very few studies aimed at improving appropriate care, and our interventions improved broader aspects of clinical management and patient endpoints. Furthermore, our evaluation on changes in physiological, intermediate outcome, endpoints (rather than rate changes) represented a more stringent test of the effect of the interventions' ability to improve patient care [26]. That the interventions changed both process and intermediate outcome further supports the utility of this method of improving patient care.

The acquisition of more clinical data than originally planned allowed the detection of a statistically significant smaller change in diastolic BP than the trial was originally powered for. Although the small effect on mean diastolic BP is unlikely to be clinically important at an individual level, it approximates to worthwhile population-level benefits. When the coefficients at ages 60 to 69 years from the Prospective Studies Collaboration are applied [27], these diastolic BP reductions produce of the order of a 5% relative reduction in stroke mortality and 3% to 4% falls in mortality from ischaemic heart disease and other vascular causes over 10 years. This mean population effect may be explained by GPs targeting action on patients above the threshold of 140/80 mmHg, as indicated by the increased odds of patients' BP being controlled in intervention practices.

The increase in HbA1c testing rates suggests that the glycaemic control message influenced practice. Our interpretation of this is that it reflects increased testing in response to increased attempts to improve glycaemic control. The absence of an effect on HbA1c levels may partly reflect the wider range of physiological and compliance issues around improving this endpoint and possible 'ceiling effects'--the mean post-intervention HbA1c for both control and intervention groups (7.5%) was close to the then QOF target level of 7.4% or less. Nonetheless, there is still scope for improvement given that 37% of patients still had levels above this target. More intensive and complex types of intervention may be required to target this problem [9].

The result that the BP message led to worse control in patients with microalbuminuria was unexpected and counterintuitive. We were underpowered for the comparison because we had not anticipated that 11 of the practices would not provide valid data, so the analysis was based on about 1,000 patients from 21 practices compared to the overall BP analysis that was based on about 7,400 patients from 34 practices. BP control over the entire period of the study was better for patients in practices randomised to receive BP messages than for those in control practices (difference in means of 3.7 and 2.7 mm Hg in systolic and diastolic BP, respectively). Thus, there was less room for improvement in practices that received the intervention. The observed effect of the intervention may be a regression to the mean phenomenon given the higher baseline values in the practices that did not receive the BP messages. There is also the possibility that there was something systematically different about the 21 practices that did and the 11 that did not contribute data to this analysis.

Taken together, our findings suggest that there may be a threshold in clinical performance beyond which prompts attached to test results do not work or have only modest effects. The review of point-of-care computer reminders did not find any specific reminder or contextual features significantly associated with effect size [12]. A review of a different behaviour change intervention, audit, and feedback found that lower baseline levels of clinical performance were associated with larger effect size [28]. Therefore, brief educational messages may still have considerable potential to improve practice and merit further exploration in at least three ways. First, their relative effects may vary across different levels of baseline performance, and it is worth actively investigating the modifying effects of baseline performance. They may work best as an initial intervention where there have been no previous efforts to improve performance and levels of appropriate performance are relatively low. Second, the impact of such interventions may be strengthened by better adapting them more specifically to clinician and patient needs [29,30]. The conditional messages (HbA1c control and ACR/BP), where a specific clinical action was recommended on the basis of a test result, were very simple and had mixed effects. Third, many clinical decisions are based upon laboratory results. The advent of real-time, interactive computerised requesting and reporting systems provides opportunities to readily identify candidate tests or conditions and then to influence practice both as requests are made and reports received, thereby allowing more efficient targeting of test request messages.

Study strengths included the use of a randomised design allowing us to be confident that any observed effects could therefore be attributed to the interventions, despite other quality improvement initiatives affecting diabetes care during the time of the study [31,32]. We are confident of fidelity given that our routine checks on the messages received revealed no deviations from randomised assignment. We used reliably coded data with minimally intrusive data collection, conducted after the intervention period was complete, that captured any effects on whole practice populations, thereby preventing any selection bias attributable to differential recruitment within randomised clusters [33]. We examined long-term outcomes (at least 24 months), thereby affording greater confidence in the sustainability of any effects.

There were several limitations. First, the study took place in one geographical area with limited patient ethnic diversity [34], so it is unclear how our results would translate into a setting with a higher ethnicity-related prevalence of diabetes. However, given that our trial involved a population of primary care practices with higher than average levels of performance for diabetes care, our estimated effects may be relatively conservative [18,24]. Second, practices on the borders of Newcastle could also conceivably have used other hospital laboratories, thereby diluting intervention effects. However, this is unlikely to be a major issue given that laboratory services are usually arranged as part of block contracts with local hospital services. Third, we do not know if the messages were actually read by clinical practice staff, especially by those responsible for acting upon results. Fourth, as we assessed one main outcome for each of four randomised interventions, we cannot rule out type 1 error as an explanation for the statistically significant effect on diastolic BP. Fifth, we did not perform an economic analysis. Whilst the intervention had low set up and negligible costs, any relative cost-effectiveness might be reduced by increased test ordering (for HbA1c), prescribing, or consultations. Finally, as an empirical intervention, we do not have any insight into how or why the intervention did or did not work. Further research is needed to understand the processes by which apparently simple interventions work.

## Conclusion

Brief educational messages attached to laboratory test results represent a simple and sustainable way to bring about improvements in care. We have demonstrated that messages aimed to improve care-produced effects on clinical practice to varying degrees, including changes in patient endpoints that may be worthwhile at a population level. These changes occurred over and above the background effects of a major pay-for-performance programme for primary care practice in a geographical area with historically high levels of performance. Given that the vast majority of studies of this type of intervention aim to decrease inappropriate test use, educational prompts aimed at improving care merits further research to identify the most appropriate clinical contexts where they can effectively target practice, explore how they work and means of enhancing their effects, and assess their cost-effectiveness.

## Competing interests

MPE is Co-Editor in Chief, RF is Deputy Editor and JG is on the Editorial Board of Implementation Science. All decisions on this manuscript were made by another editor.

## Authors' contributions

MPE conceived the project. RF was principal investigator. All authors contributed to the design of the study. SH, RF, and IG were responsible for running the project. NS was responsible for the statistical analyses. All authors interpreted the data and findings. RF wrote the first draft of the manuscript, all authors commented on it and all further revisions. All authors read and approved the final manuscript. RF is guarantor for the paper.
